# The Bergen Shopping Addiction Scale: reliability and validity of a brief screening test

**DOI:** 10.3389/fpsyg.2015.01374

**Published:** 2015-09-17

**Authors:** Cecilie S. Andreassen, Mark D. Griffiths, Ståle Pallesen, Robert M. Bilder, Torbjørn Torsheim, Elias Aboujaoude

**Affiliations:** ^1^Department of Psychosocial Science, University of Bergen, Bergen, Norway; ^2^The Competence Centre, Bergen Clinics Foundation, Bergen, Norway; ^3^International Gaming Research Unit, Nottingham Trent University, Nottingham, UK; ^4^Norwegian Competence Center for Sleep Disorders, Bergen, Norway; ^5^Semel Institute for Neuroscience and Human Behavior, University of California, Los Angeles, Los Angeles, CA, USA; ^6^OCD Clinic, Department of Psychiatry and Behavioral Sciences, Stanford University School of Medicine, Stanford, CA, USA

**Keywords:** assessment, compulsive buying, personality, psychological distress, psychometrics, scale, self-esteem, shopping addiction

## Abstract

Although excessive and compulsive shopping has been increasingly placed within the behavioral addiction paradigm in recent years, items in existing screens arguably do not assess the core criteria and components of addiction. To date, assessment screens for shopping disorders have primarily been rooted within the impulse-control or obsessive-compulsive disorder paradigms. Furthermore, existing screens use the terms ‘shopping,’ ‘buying,’ and ‘spending’ interchangeably, and do not necessarily reflect contemporary shopping habits. Consequently, a new screening tool for assessing shopping addiction was developed. Initially, 28 items, four for each of seven addiction criteria (salience, mood modification, conflict, tolerance, withdrawal, relapse, and problems), were constructed. These items and validated scales (i.e., Compulsive Buying Measurement Scale, Mini-International Personality Item Pool, Hospital Anxiety and Depression Scale, Rosenberg Self-Esteem Scale) were then administered to 23,537 participants (*M*_age_ = 35.8 years, *SD*_age_ = 13.3). The highest loading item from each set of four pooled items reflecting the seven addiction criteria were retained in the final scale, The Bergen Shopping Addiction Scale (BSAS). The factor structure of the BSAS was good (RMSEA = 0.064, CFI = 0.983, TLI = 0.973) and coefficient alpha was 0.87. The scores on the BSAS converged with scores on the Compulsive Buying Measurement Scale (CBMS; 0.80), and were positively correlated with extroversion and neuroticism, and negatively with conscientiousness, agreeableness, and intellect/imagination. The scores of the BSAS were positively associated with anxiety, depression, and low self-esteem and inversely related to age. Females scored higher than males on the BSAS. The BSAS is the first scale to fully embed shopping addiction within an addiction paradigm. A recommended cutoff score for the new scale and future research directions are discussed.

## Introduction

The latest (fifth) edition of the *Diagnostic and Statistical Manual for Mental Disorders* (DSM-5; American Psychiatric Association, 2013) included three noticeable changes from the DSM-IV that have implications for researchers in the field of behavioral addiction: (i) the section Substance-Related Disorders (American Psychiatric Association, 1994) was renamed “Substance-Related and Addictive Disorders," (ii) Gambling Disorder was moved from the Impulse-Control Disorders Section (American Psychiatric Association, 1994) to the Substance-Related and Addictive Disorders section and classified as a behavioral addiction, and (iii) Internet Gaming Disorder was introduced (in Section 3 ‘Emerging Measures and Models’; American Psychiatric Association, 2013). Together, these changes represent an increasing recognition of non-chemical addictions. However, at present, most non-chemical addictions are not yet embedded in the psychiatric nosology. This includes shopping addiction, despite this disorder having been recognized in the psychiatric literature for over a century (Kraepelin, 1915).

Whether compulsive and excessive shopping represents an impulse-control, obsessive-compulsive, or addictive disorder has been debated for several years (Aboujaoude, 2014; Piquet-Pessôa et al., 2014). This fact is reflected in the many names that have been given to this problem, including “oniomania,” “shopaholism,” “compulsive shopping,” “compulsive consumption,” “impulsive buying,” “compulsive buying” and “compulsive spending” (Aboujaoude, 2014; Andreassen, 2014). Andreassen (2014, p. 198) recently argued that shopping disorder would be best understood from an addiction perspective, defining it as “being overly concerned about shopping, driven by an uncontrollable shopping motivation, and to investing so much time and effort into shopping that it impairs other important life areas.” Several authors share this view (e.g., Albrecht et al., 2007; Davenport et al., 2012; Hartston, 2012), as a growing body of research shows that those with problematic shopping behavior report specific addiction symptoms such as craving, withdrawal, loss of control, and tolerance (Black, 2007; Workman and Paper, 2010). For the most part, this empirical research also suggests that the typical shopping addict is young, female, and of lower educational background (Black, 2007; Davenport et al., 2012; Maraz et al., 2015b). Research has also linked those with problematic shopping to individual characteristics typical for other addictive behaviors (Aboujaoude, 2014).

Some of this research has involved the five-factor model of personality (Costa and McCrae, 1992; Wiggins, 1996). Extroversion has been positively associated with shopping addiction (Balabanis, 2002; Mikolajczak-Degrauwe et al., 2012; Andreassen et al., 2013; Thompson and Prendergast, 2015), suggesting that extroverts may be using shopping to uphold their social status and sustain their social attractiveness, such as by buying a new outfit and accessories for every occasion. Neuroticism has also been consistently been related to shopping addiction (Wang and Yang, 2008; Mikolajczak-Degrauwe et al., 2012; Andreassen et al., 2013; Thompson and Prendergast, 2015). Neurotic individuals, typically being anxious, depressive, and self-conscious may use shopping as means of reducing their negative emotional feelings.

Conscientiousness, on the other hand, appears to be a protective factor (Mowen and Spears, 1999; Wang and Yang, 2008; Andreassen et al., 2013). People with low conscientiousness scores appear to shop due to low ability to be structured and responsible (Andreassen et al., 2013). Also, the relationship between agreeableness and shopping addiction appears to be more ambivalent. Some studies have reported a positive relationship (Mowen and Spears, 1999; Mikolajczak-Degrauwe et al., 2012), while others a negative one (Balabanis, 2002; Andreassen et al., 2013). High degrees of agreeableness may represent a protective factor for developing shopping addiction (or addiction of any kind), as such individuals typically avoid conflicts and disharmony. Since addictive behaviors often create conflicts with others, it seems reasonable that shopping addiction would be negatively related to agreeableness. At the same time, agreeable people may be more prone to fall for exploitative marketing techniques since they easily trust others. Finally, the openness to experience trait has typically been unrelated to shopping addiction (Mowen and Spears, 1999; Wang and Yang, 2008; Andreassen et al., 2013). However, at least two studies have reported a negative relationship (Balabanis, 2002; Mikolajczak-Degrauwe et al., 2012), suggesting that shopping addicts are less adventurous and less curious and put less emphasis on abstract thinking than their counterparts.

Addictive behaviors may also be related to individual differences in self-esteem and psychological distress. Empirical research has consistently reported significantly lower levels of self-esteem among shopping addicts (Davenport et al., 2012; Maraz et al., 2015b). Such findings suggest that irrational beliefs such as “buying a product will make life better” and “shopping this item will enhance my self-image” may trigger excessive shopping behavior in people with low self-esteem (McQueen et al., 2014; Roberts et al., 2014; Harnish and Bridges, 2015). However, this may be related to depression, which has been shown to be highly comorbid with problematic shopping (Aboujaoude, 2014). In line with this, psychological distress such as anxiety has also often been associated with shopping addiction (Otero-López and Villardefrancos, 2014; Roberts et al., 2014; Maraz et al., 2015b). It has also been suggested that self-critical people shop in order to escape, or cope with, negative feelings (Rick et al., 2014; Weinstein et al., 2015). As existing research is primarily based on cross-sectional studies, we know little about the directionality of these relationships. Consequently, preexisting psychological distress may lead to shopping addiction, or vice versa (Müller et al., 2014; Lieb, 2015). In this regard, it should be noted that shopping addiction has been explained as a way of regulating neurochemical (e.g., serotonergic, dopaminergic, opioid) abnormalities and has been successfully treated with pharmacological agents, including selective serotonin reuptake inhibitors (SSRIs) and opioid antagonists – in line with other behavioral addictions (Potenza and Hollander, 2002). To that effect, an fMRI-study reported a significant difference in activation of reward and pain circuit systems between shopping addicts and non-shopping addicts during purchasing decisions (Raab et al., 2011). Therefore, an argument could also be made for a biological basis for this condition.

A limitation of prior research is the lack of a common understanding about how problematic shopping should be defined, conceptualized, and measured (Manolis and Roberts, 2008; Aboujaoude, 2014; Piquet-Pessôa et al., 2014). This leaves us with unreliable prevalence estimates ranging from 1 to 20% and beyond (Dittmar, 2005; Koran et al., 2006; MacLaren and Best, 2010; Sussman et al., 2011; Otero-López and Villardefrancos, 2014; Maraz et al., 2015a). Although several scales for assessing shopping addiction have been developed, mainly in the late 1980s and early 1990s, many have poor theoretical anchoring and/or are primarily rooted within the impulse-control paradigm (Andreassen, 2014; Maraz et al., 2015a). In addition, several items of existing scales appear outdated with regards to modern consumer patterns. For example, some of the most widely used scales, the Compulsive Buying Scale, includes somewhat outdated items such as “I wrote a check” (Faber and O’Guinn, 1992) or “when I enter a shopping center” (Valence et al., 1988). Shoppers rarely use checks any more, and in contemporary society, many favor online over oﬄine shopping. Another limitation is that the existing scales are relatively lengthy. Koronczai et al. (2011) noted that a suitable measure should meet key criteria including brevity, so that it can be used for impulsive individuals and be included in time-limited surveys.

Although two new scales have been developed more recently (Christo et al., 2003; Ridgway et al., 2008), they do not approach problematic shopping behavior as an addiction in terms of core addiction criteria (i.e., salience, mood modification, tolerance, withdrawal, conflict, relapse, and resulting problems) that have been emphasized in several behavioral addictions (e.g., Brown, 1993; Griffiths, 1996, 2005; Lemmens et al., 2009; Andreassen et al., 2012). More specifically, these criteria involve obsessing over shopping/buying activities; shopping/ buying as a way to enhance feelings; the need to increase the behavior over time in order to feel satisfied; distress if the behavior is reduced or eliminated; ignoring other activities because of the behavior; unsuccessful attempts to control or reduce the behavior; and suffering direct or indirect harm as a result of the behavior. Existing problematic shopping scales typically involve one or several of these symptoms, but fail to fully incorporate them all. In addition, since new Internet-related technologies can greatly facilitate the emergence of problematic shopping behavior because of factors such as accessibility, affordability, anonymity, convenience, and disinhibition (Wang and Yang, 2008; Widyanto and Griffiths, 2010; Aboujaoude, 2011), there is a need for a psychometrically robust instrument that assesses problematic shopping across all platforms.

Given this background, a shopping addiction scale [the Bergen Shopping Addiction Scale (BSAS)] was developed, containing a small number of items that reflect the seven aforementioned elements of addiction, thus ensuring its content validity in an addiction framework. It was hypothesized that the new shopping addiction scale would correlate highly with measures of similar constructs (convergent validity) and less with measures of more divergent or unrelated constructs (discriminant validity; Nunnally and Bernstein, 1994). Accordingly, the following hypotheses were investigated: (H1) the BSAS will have a one-dimensional factor structure with high factor loading (>0.60) for all items, and with fit indexes [root mean square error of approximation (RMSEA), comparative fit index (CFI), and Tucker–Lewis Index (TLI)] showing good fit with the data; (H2) the internal consistency (Cronbach’s alpha) of the BSAS will be high (>0.80); (H3) scores on BSAS will correlate positively and significantly with scores on the CBMS (Valence et al., 1988); (H4) BSAS scores will be positively associated with being female and will be inversely related to age; (H5) the scores on the BSAS will be positively associated with extroversion and neuroticism, but negatively associated with conscientiousness and agreeableness, and unrelated to intellect/imagination; (H6) finally, scores on the BSAS will be positively related to anxiety and depression, and negatively to self-esteem.

## Materials and Methods

### Sample

The sample comprised 23,537 participants (15,301 females and 8,236 males). The mean age was 35.8 years (*SD* = 13.3). Two-thirds (65.3%) were married or partnered, and 34.7% were single. Educational level ranged from primary school (10.0%), to high school (25.3%), vocational school (17.0%), bachelor’s degree (32.4%), master’s degree (14.2%), and Ph.D. (1.2%). In terms of occupational status, 4,962 were students, 12,967 worked full-time, 3,515 worked part-time, 651 were retired, 390 were homemakers, 1,113 were on permanent disability pension, 1,147 were receiving temporary rehabilitation benefits, 541 were unemployed, and 407 reported “other.” Some students/workers ticked boxes for being both a student as well as employed.

### Procedure

In the first stage of scale development, 28 potential items were included. The scale was constructed based on the seven basic components of addiction originally proposed by Brown (1993) and developed and modified by Griffiths (1996). Four items for each component were constructed. Since addiction was considered a main construct comprising seven different components, a second-order model was initially set up, with addiction (i.e., buying addiction) as a second-order construct and where the seven different components comprised the first-order factors. For some items the wording was similar to that used in the diagnostic criteria for pathological gambling (American Psychiatric Association, 2013) and the Game Addiction Scale (Lemmens et al., 2009). The 28 items were included in a self-report questionnaire with additional questions about a person’s demographics, compulsive buying habits, personality, self-esteem, and symptoms of anxiety, and depression. The questions were distributed via the online edition of five nationwide newspapers in Norway in March, April and May 2014. The survey was available online for 1 day up to 1 week on the various newspaper websites. Information about the study purpose was provided immediately after participants clicked the survey link. Consent to participate was deemed as given since participants completed the questionnaire. Also, after survey completion, participants were provided interactive feedback on their shopping habits. No other incentives were offered in return for participation. All questions were collected anonymously and no interventions were made. Participant responses were stored by an Internet survey agency before being passed over to the research team. Respondents that only clicked on the link or gave only a few answers where deleted from the data file (*n* = 18,433). The study was carried out in accordance with the Helsinki Convention and the Norwegian Health Research Act.

### Instruments

#### Demographics

Participants were asked for information about their age, gender, level of education, relationship, and occupational status.

#### The Bergen Shopping Addiction Scale

In order to develop a new, brief, updated shopping addiction scale, a pool of 28 items were first created. This pool was based on the seven addiction criteria outlined earlier in the paper. Four items for each addiction criterion were constructed based upon diagnostic criteria for pathological gambling (American Psychiatric Association, 2013), the Game Addiction Scale (Lemmens et al., 2009) and a general literature review of common symptoms associated with shopping and buying addiction. The response options were *completely disagree* (0), *disagree* (1), *neither disagree nor agree* (2), *agree* (3), and *completely agree* (4) (see **Table 1**). Higher scores indicate higher levels of shopping addiction. The best items related to each addiction criterion were retained in the final scale (see ‘Statistics’ section below for more detail on individual items).

**Table 1:** Initial pool items for the shopping addiction scale^1^.

No.	Dimension	Item text
1	Salience	Shopping/buying is the most important thing in my life
2	Salience	I think about shopping/buying things all the time^2^
3	Salience	I spend a lot of time thinking of or planning shopping/buying
4	Salience	Thoughts about shopping/buying keep popping in my
5	Mood modification	I shop in order to feel better
6	Mood modification	I shop/buy things in order to change my mood^2^
7	Mood modification	I shop/buy things in order to forget about personal problems
8	Mood modification	I shop/buy things in order to reduce feelings of guilt, anxiety, helplessness, loneliness, and/or depression
9	Conflict	I shop/buy so much that it negatively affects my daily obligations (e.g., school and work)^2^
10	Conflict	I give less priority to hobbies, leisure activities, job/studies, or exercise because of shopping/buying
11	Conflict	I have ignored love partner, family, and friends because of shopping/buying
12	Conflict	I often end up in arguments with other because of shopping/buying
13	Tolerance	I feel an increasing inclination to shop/buy things
14	Tolerance	I shop/buy much more than I had intended/planned
15	Tolerance	I feel I have to shop/buy more and more to obtain the same satisfaction as before^2^
16	Tolerance	I spend more and more time shopping/buying
17	Relapse	I have tried to cut down on shopping/buying without success
18	Relapse	I have been told by others to reduce shopping/buying without listening to them
19	Relapse	I have decided to shop/buy less, but have not been able to do so^2^
20	Relapse	I have managed to limit shopping/buying for periods, and the experienced relapse
21	Withdrawal	I become stressed if obstructed from shopping/buying things
22	Withdrawal	I become sour and grumpy if I for some reasons cannot shop/buy things when I feel like it
23	Withdrawal	I feel bad if I for some reason are prevented from shopping/buying things^2^
24	Withdrawal	I there has been a while since I last shopped I feel a strong urge to shop/buy tings
25	Problems	I shop/buy so much that it has caused economic problems
26	Problems	I shop/buy so much that it has impaired my well-being^2^
27	Problems	I have worried so much about my shopping problems that it sometimes has made me sleepless
28	Problems	I have been bothered with poor conscience because of shopping/buying


*^1^The instruction was: for each item tick the response alternative (ranging from ≪completely disagree≫ to ≪completely agree≫) that best describes you. The statements relate to your thoughts, feelings and actions the last 12 months.*

*^2^Item retained in the final scale.*

#### Compulsive Buying Measurement Scale

This scale comprises 13 items for assessing compulsive buying tendencies (Valence et al., 1988; Scherhorn et al., 1990). Items are answered on a 5-point scale anchored from *Strongly disagree* (1) to *Strongly agree* (5) (e.g., “When I have money, I cannot help but spend part or all of it”). Higher scores reflect more compulsive buying. The CBMS, initially a pool of 16 impulse-control based items, was the first quantitative measure of compulsive buying tendencies. It is brief, suitable for adolescents and adults, widely used, and exists in several languages. Although the CBMS has proven reliable and valid, it has also been criticized for being outdated (Andreassen, 2014). See **Table 2** for scale characteristics for the present study sample, including Cronbach’s alphas.

**Table 2:** Descriptive data and correlation coefficients between study variables (*N* = 23,535–23,537).

Variables	1	2	3	4	5	6	7	8	9	10
(1) Shopping addiction	–									
(2) Compulsive buying	0.80^∗∗^	–								
(3) Extroversion	0.03^∗∗^	0.05^∗∗^	–							
(4) Agreeableness	0.04^∗∗^	0.07^∗∗^	0.30^∗∗^	–						
(5) Conscientiousness	-0.11^∗∗^	-0.18^∗∗^	0.09^∗∗^	0.13^∗∗^	–					
(6) Neuroticism	0.30^∗∗^	0.31^∗∗^	-0.10^∗∗^	-0.09^∗∗^	-0.16^∗∗^	–				
(7) Intellect/imagination	-0.03^∗∗^	-0.01	0.16^∗∗^	0.12^∗∗^	-0.12^∗∗^	-0.00	–			
(8) Anxiety	0.34^∗∗^	0.34^∗∗^	-0.12^∗∗^	0.03^∗∗^	-0.23^∗∗^	0.64^∗∗^	0.03^∗∗^	–		
(9) Depression	0.19^∗∗^	0.18^∗∗^	-0.30^∗∗^	-0.23^∗∗^	-0.26^∗∗^	0.42^∗∗^	-0.08^∗∗^	0.55^∗∗^	–	
(10) Self-esteem	-0.26^∗∗^	-0.27^∗∗^	0.32^∗∗^	0.06^∗∗^	0.30^∗∗^	-0.53^∗∗^	0.11^∗∗^	-0.56^∗∗^	-0.55^∗∗^	–
*M*	3.01	23.90	13.47	16.32	14.90	11.81	14.26	6.64	4.10	29.23
*SD*	4.32	10.23	3.65	2.95	3.22	3.54	3.14	3.92	3.20	5.34
Range	0–28	18–90	4–16	4–16	4–16	4–16	4–16	0–21	0–21	10–40
Alpha	0.87	0.91	0.81	0.76	0.70	0.73	0.69	0.82	0.75	0.89
Items	7	13	4	4	4	4	4	7	7	10


*^∗∗^*p* < 0.01.*

#### Mini-International Personality Item Pool (Mini-IPIP)

This scale comprises 20 items for assessing personality (Donnellan et al., 2006). Four items reflect each of the personality traits of the established Five-Factor Model of personality (Costa and McCrae, 1992; Wiggins, 1996): extraversion (e.g., “Am the life of the party”), agreeableness (e.g., “Sympathize with other’s feelings”), conscientiousness (e.g., “Get chores done right away”), neuroticism (e.g., “Have frequent mood swings”), and intellect/imagination (e.g., “Have a vivid imagination”), the latter being equal to the openness dimension. All items are answered on a 5-point scale ranging from *Very inaccurate* (1) to *Very accurate* (5). Studies have shown acceptable psychometric properties of this personality measure (e.g., Donnellan et al., 2006; Andreassen et al., 2013). Relatively low alpha values (<0.70) have also been reported for some of the subscales (Andreassen et al., 2014).

#### Hospital Anxiety and Depression Scale (HADS)

This 14-item scale comprises seven items assessing anxiety and another seven items assessing depression (Zigmond and Snaith, 1983). All items are answered along a 4-point frequency scale ranging from 0 to 3. However, the frequency categories are different for almost each question asked. For example, for the item “I feel tense and wound up” the alternatives range from *Not at all* (0), to *From time to time, occasionally* (1), *A lot of the time* (2), *Most of the time* (3); and for “I feel as if I am slowed down” the categories are *Not at all* (0), *Sometimes* (1), *Very often* (2), *Nearly all the time* (3). The Hospital Anxiety and Depression Scale (HADS) has shown good validity in clinical populations as well as in the general population (Bjelland et al., 2002).

#### Rosenberg Self-Esteem Scale

This scale comprises 10 items for assessing levels of self-esteem (Rosenberg, 1965). Items are answered on a 4-point scale using anchors of *Strongly agree* (0) and *Strongly disagree* (3) (e.g., “I feel I do not have much to be proud of”). The higher the score, the higher the self-esteem. A recent meta-analysis provided good support for its factor structure and psychometric qualities (Huang and Dong, 2012).

### Statistics

Descriptive statistics including frequencies, means, standard deviation and Cronbach alphas were calculated for all study variables (see **Table 2**). To identify the best item to include in the final scale, a second-order confirmatory factor analysis was conducted in a randomly selected half of the sample (*n* = 11,768). The second-order factor comprised shopping addiction whereas the seven first order factors (Salience, Mood modification, Conflict, Tolerance, Relapse, Withdrawal and Problems) were each reflected by four items. The item with the highest loading on each associated addiction criterion using confirmatory factor analysis was deemed the best item and was the item that was retained in the final scale that was tested in the other half of the sample (*n* = 11,769). The fit of these models was investigated by confirmatory factor analyses using AMOS, version 21.0. In the final model, correlations between error terms were allowed providing this had substantive meaning (Byrne, 2010). The RMSEA, the CFI and the TLI were used as fit indexes. As a general rule, for a model with acceptable fit to the data, the three indexes should be <0.08, >0.90, and >0.90, respectively, whereas the three corresponding values for a good fit would be <0.06, >0.95, and 0.95, respectively (Hu and Bentler, 1999). The final scale was further investigated by Cronbach alpha and corrected item-total correlations.

In order to investigate the convergent validity of the new scale, the zero-order correlation with the CBMS (Valence et al., 1988) was calculated. Finally, for investigating the convergent as well as the discriminative validity of the new scale, a hierarchical regression analysis was conducted where the new scale (BSAS) comprised the dependent variable. The predictors included in the first step were gender, age, and marital status. In the second and final step, symptoms of depression, anxiety, and self-esteem were included, as well as the measures of the five-factor model of personality.

## Results

### Scale Construction

**FIGURE 1:**
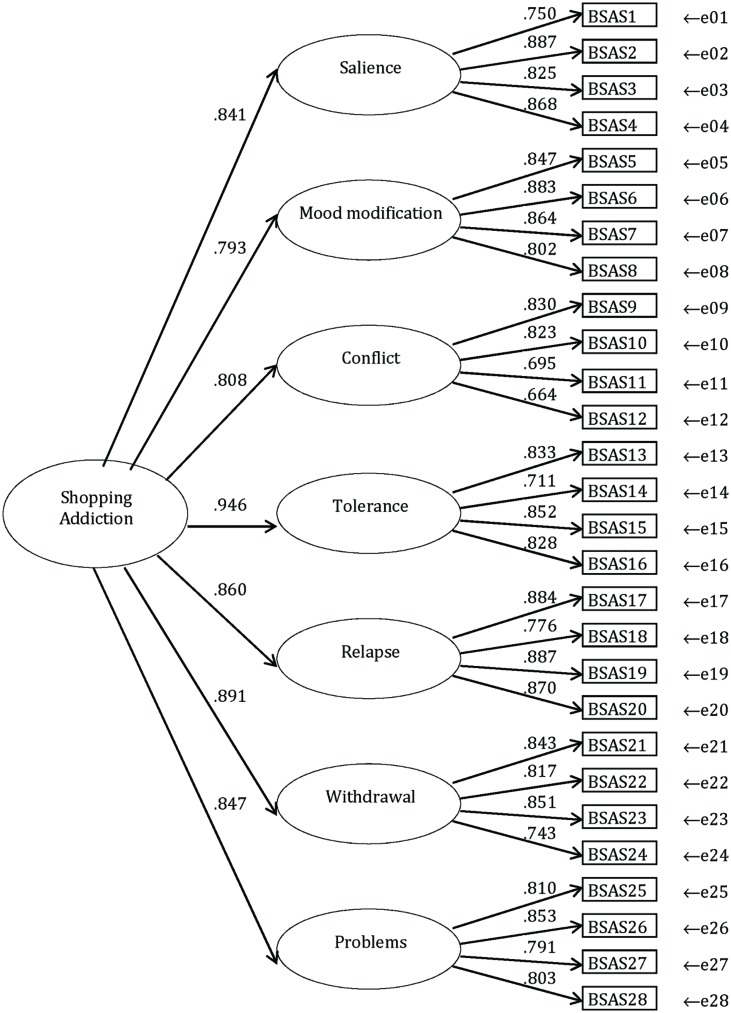
**Second-order factor structure of the 28 shopping addiction items (*n* = 11,768) showing standardized factor loadings**.

The second-order factor structure is shown in **Figure 1**. The standardized second-order factor loadings ranged from 0.793 (‘mood modification’) to 0.946 (‘tolerance’). The highest first order loading for each of the seven factors ranged from 0.830 (Item 9 on ‘conflict’) to 0.887 (Item 2 on ‘salience’). All loadings were significant (*p* < 0.001). The second-order model had acceptable fit with the data, χ^2^(df = 343, *n* = 11,768) = 25324, CFI = 0.909, RMSEA = 0.079 (90% CI = 0.078–0.079), TLI = 0.899. A one-factor model where all 28 items loaded on one factor had poorer fit [χ^2^(df = 350, *n* = 11,768) = 70529, CFI = 0.743, RMSEA = 0.131 (90% CI = 0.130–0.131), TLI = 0.723] with the data than the second-order factor model.

**FIGURE 2:**
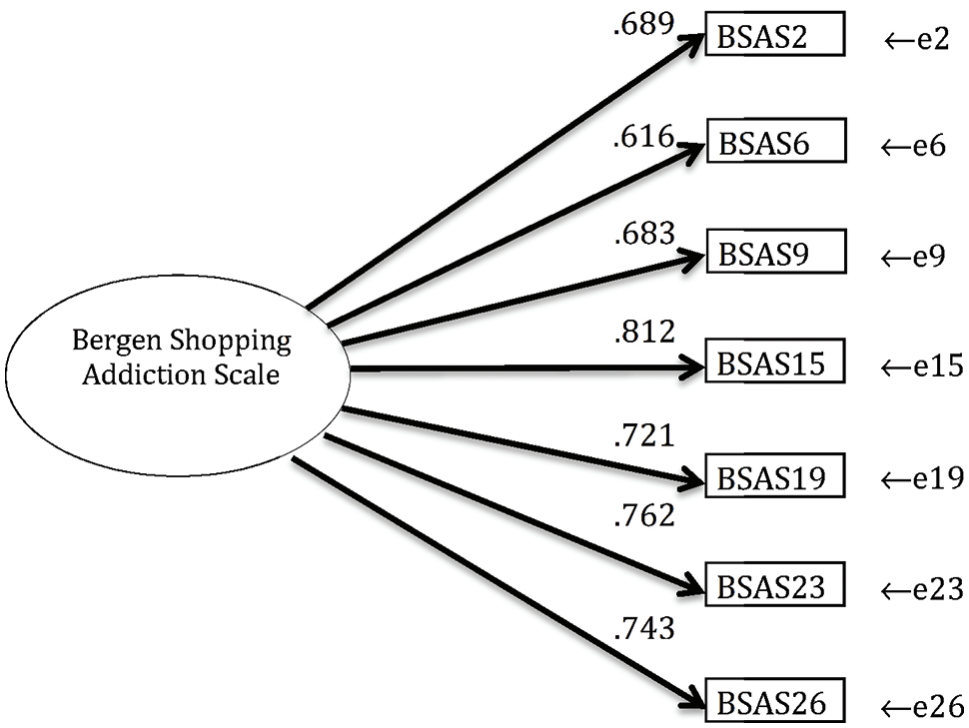
The final factor structure of Bergen Shopping Addiction Scale (*n* = 11,769) showing standardized factor loadings.

The factor structure of the final BSAS is shown in **Figure 2**. The standardized factor loadings ranged from 0.616 to 0.812. All loadings were significant (*p* < 0.001). The error terms of the two first items were allowed to correlate as they both reflected inner thoughts or states. The correlation coefficient was 0.280. The model had good fit with the data, χ^2^(df = 13, *n* = 11,769) = 639, CFI = 0.983, RMSEA = 0.064 (90% CI = 0.060–0.068), TLI = 0.973. In terms of measurement invariance across gender, the data indicated configural invariance [χ^2^ (df = 26, *N* = 23,537) = 1249, CFI = 0.983, RMSEA = 0.045 (90% CI = 0.043–0.047), TLI = 0.973]. In order to test invariance (whole sample), the ΔCFI was used and should be below 0.01 for not rejecting the null hypothesis of invariance (Cheung and Rensvold, 2002). In the present study, the ΔCFI between the unconstrained model and a model with constraints on measurement weights was 0.003, thus showing metric invariance across gender.

### Psychometric Properties of the Bergen Shopping Addiction Scale

Cronbach’s alpha of the BSAS was 0.867. The mean inter-item correlation coefficient was 0.43. The corrected item-total correlation coefficients for BSAS2, BSAS6, BSAS9, BSAS15, BSAS19, BSAS23, and BSAS26 (comprising the final scale) were 0.692, 0.622, 0.615, 0.742, 0.658, 0.692, and 0.661, respectively.

### Convergent and Discriminative Validity

The correlation coefficient between the composite score of the BSAS and the Compulsive Buying Scale was 0.798. Furthermore, both scales showed similar correlation patterns with the other study variables (see **Table 2**).

### Relations with Demographics, Personality, Anxiety, Depression, and Self-Esteem

The results from the hierarchical multiple linear regression analysis showed that demographic variables (i.e., age, gender, marital status) explained 8.2% of the variance in the scores on the BSAS at Step 1 (*F*_3,23531_ = 700.40, *p* < 0.001) (see **Table 3**). Age and gender contributed significantly. After entry of all independent variables at Step 2 (Δ*F*_8,23523_ = 355.76, *p <* 0.001), age (β = -0.090) and gender (β = 0.204) still contributed significantly, along with extroversion (β = 0.096), agreeableness (β = -0.046), conscientiousness (β = -0.048), neuroticism (β = 0.085), intellect/imagination (β = -0.026), anxiety (β = 0.197), depression (β = 0.036), and self-esteem (β = -0.049). Taken as a whole, the model explained 18.1% (*F*_11,23523_ = 472.79, *p* < 0.001) of the variance in shopping addiction.

**Table 3:** Results from the hierarchical regression analysis where age, gender, marital status, Big Five traits, anxiety, depression, and self-esteem were regressed upon the Bergen Shopping Addiction Scale score (*N* = 23,537).

	*B*	*SE*	*β*	*t*	Δ*R*^2^
**Step 1**					0.082^∗∗^
Age	-0.052	0.002	-0.159	-24.813^∗∗∗^	
Gender (1 = ♂, 2 = ♀)	2.202	0.057	0.243	38.826^∗∗∗^	
Marital status^a^	0.012	0.058	0.001	0.207	
**Step 2**					0.099^∗∗^
Age	-0.029	0.002	-0.090	-14.378^∗∗∗^	
Gender (1 = ♂, 2 = ♀)	1.851	0.061	0.204	30.529^∗∗∗^	
Marital status^a^	-0.067	0.056	-0.007	-1.198	
Extroversion	0.113	0.008	0.096	14.456^∗∗∗^	
Agreeableness	-0.068	0.010	-0.046	-6.871^∗∗∗^	
Conscientiousness	-0.064	0.009	-0.048	-7.339^∗∗∗^	
Neuroticism	0.104	0.010	0.085	10.377^∗∗∗^	
Intellect/imagination	-0.036	0.009	-0.026	-4.232^∗∗∗^	
Anxiety	0.217	0.010	0.197	22.425^∗∗∗^	
Depression	0.048	0.011	0.036	4.410^∗∗∗^	
Self-esteem	-0.040	0.007	-0.049	-5.825^∗∗∗^	


*B = unstandardized regression coefficient, standardized regression coefficient;*

*^∗∗∗^*p* < 0.001; ^a^Marital status: in a relationship = 1, not in a relationship = 2.*

## Discussion

The present study developed a new instrument to assess shopping addiction and examined its psychometric properties within a large sample of Norwegian individuals. Although excessive and problematic shopping behavior has beive behavior, previous screening instruments have failed to capture core addiction criteria. The new BSAS, based on contemporary addiction theory and criteria, addressed these shortcomings.

The first hypothesis concerned the scale construction of BSAS. All loadings in the final scale were above 0.60 and significant. The CFI was above 0.98, the RMSEA was 0.06, and the TLI was 0.97 – indicating acceptable to good fit (Hu and Bentler, 1999). Thus, the first hypothesis of a one-dimensional factor structure of the final scale that showed good fit with the data was supported.

The second hypothesis targeted scale psychometrics. The internal consistency was satisfactory (α = 0.87), mean inter-item correlation was 0.43, and the corrected item-total correlation coefficients for the seven items ranged from 0.62 to 0.74. These finding further supported the one-factor structure of the scale. Consequently, the second hypothesis was also supported by a psychometric analysis of the data.

The third hypothesis focused on convergent validity of BSAS. As expected, the scores on the new scale correlated highly (0.80) with the scores of the CBMS (Valence et al., 1988). Furthermore, the two scales showed similar correlational patterns with the other study variables. The high correlation between the BSAS and CBMS attest to the convergent validity of the new scale and provided sufficient confirmatory support for the third hypothesis.

Scores on the BSAS were significantly higher among females, as well as being inversely related to age. This is in line with previous research (Black, 2007; Davenport et al., 2012; Maraz et al., 2015b), and the present study’s fourth hypothesis – showing that basic demographic variables explain some of the variance in shopping addiction. Hence, shopping addiction seems to be more predominant in females, although some scholars and large surveys have disputed such a finding (Koran et al., 2006). Also, recent study of compulsive buying among Internet shoppers reported there were no gender differences (Weinstein et al., 2015). However, this may simply indicate that more men prefer shopping online to shopping oﬄine because it is convenient, comfortable, and anonymous. The differences in motivation and subsequent problematic use between online and oﬄine shopping should be investigated in more detail in future studies. The findings of the present study are also in accord with previous studies demonstrating that shopping addiction is typically initiated in late adolescence and emerging adulthood, and that it appears to decrease with age. This has been hypothesized to reflect maturational changes in frontal cortical and subcortical mono-aminergic systems, making adolescents and young adults more vulnerable than older individuals to develop and maintain addictions (Chambers et al., 2003) and is also in line with studies demonstrating biological correlates to shopping addiction (Raab et al., 2011). The fourth hypothesis was therefore supported by the findings.

The fifth and sixth hypotheses concerned discriminative validity of the BSAS, implying that scores would be significantly linked to individual characteristics in terms of key personality traits and symptoms of psychological distress. The fifth hypothesis expected scores on the BSAS to be positively associated with extroversion and neuroticism, but negatively associated with conscientiousness and agreeableness, and unrelated to intellect/imagination. In agreement with the hypothesis and previous studies (e.g., Balabanis, 2002; Mikolajczak-Degrauwe et al., 2012; Andreassen et al., 2013; Thompson and Prendergast, 2015), a positive association between shopping addiction and extroversion was found. This association may reflect that, in general, extroverts need more stimulation than non-extroverted individuals, a notion that is in line with studies showing that extroversion is associated with addictions more generally (e.g., Hill et al., 2000). The present finding may also reflect the notion that extroverts purchase specific types of products excessively as a means to express their individuality, enhance personal attractiveness, or as a way to belong to a certain privileged group a (e.g., the buying of high end luxury goods; Verplanken and Herabadi, 2001; Aboujaoude, 2014).

Agreeableness was positively related to shopping addiction in the correlation analysis. However, after controlling for demographic factors in the regression analysis, agreeableness was negatively associated with shopping addiction, a finding that is in line with some previous studies (e.g., Balabanis, 2002; Andreassen et al., 2013). Still, it cannot be ruled out that the latter finding represents a suppressor effect (MacKinnon et al., 2000) and exploratory analysis indicated that inclusion of gender compared to the other independent variables in the regression analysis caused the greatest increase of the regression coefficient of agreeableness (results not shown). It has been proposed that agreeableness (in general) is a protective factor against the development of addictions, since addictions normally cause interpersonal conflicts (Andreassen et al., 2013). Therefore, the findings of the present study concerning agreeableness support Hypothesis 5.

As expected, shopping addiction was positively associated with neuroticism. This may be because neuroticism is a general vulnerability factor for the development of psychopathology (Winter and Kuiper, 1997) and that people scoring high on neuroticism engage excessively in different behaviors in order to escape from dysphoric feelings (O’Brien and DeLongis, 1996). Conscientiousness, on the other hand, was inversely related to shopping addiction. This is also in line with Hypothesis 5 and is consistent with findings from previous studies (e.g., Mowen and Spears, 1999; Wang and Yang, 2008; Andreassen et al., 2013). This can be explained by behaviors that typically characterize people with high scores on conscientiousness such as good planning ability (Verplanken and Herabadi, 2001), high self-control, and the ability to resist temptations (Wang and Yang, 2008).

It was also expected that intellect/imagination would be unrelated to shopping addiction. However, contrary to Hypothesis 5, this trait was negatively associated with shopping addiction. Although the findings from the present study did not support the hypothesis, a negative association between intellect/imagination and excessive shopping has been reported in the literature previously (e.g., Mikolajczak-Degrauwe et al., 2012). This probably reflects that people scoring high on this trait are intellectually curious and as such have a somewhat better perception of reality which prevents them from engaging in shopping addiction (Mikolajczak-Degrauwe et al., 2012). Another explanation is that shopping can be regarded as a conventional activity, which is at odds with central features of the openness/intellect trait such as imagination, curiosity, and unconventional values (Costa and Widiger, 2002). Overall, we conclude that Hypothesis 5 was supported for four out of the five five-factor model traits.

The sixth and final hypothesis was that shopping addiction would be positively related with symptoms of depression and anxiety and negatively related to self-esteem. Both scores on depression and anxiety were positively associated with the scores on shopping addiction in the present study. These findings are in line with previous studies (e.g., Otero-López and Villardefrancos, 2014; Rick et al., 2014; Roberts et al., 2014; Maraz et al., 2015b; Weinstein et al., 2015). Here, shopping may function as an escape mechanism for dysphoric feelings of anxiety and depression (DeSarbo and Edwards, 1996; Rick et al., 2014), or, conversely excessive shopping may cause anxiety and depression (e.g., fear and sadness related to the consequences; Roberts, 1998). Both notions are consistent with the present finding.

The present study also found that the scores of shopping addiction were inversely related to the scores on self-esteem. This is in keeping with the findings of previous studies (e.g., Davenport et al., 2012; Maraz et al., 2015b) and implies that some individuals shop excessively in order to obtain higher self-esteem (e.g., associated “rub-off” effects from high status items such as popularity, compliments, in-group ‘likes,’ omnipotent feelings while buying items, attention during the shopping process from helping retail personnel; McQueen et al., 2014; Roberts et al., 2014; Harnish and Bridges, 2015), to escape from feelings of low self-esteem (DeSarbo and Edwards, 1996), or that shopping addiction lowers self-esteem (Rodrigues-Villarino et al., 2006). It is concluded that Hypothesis 6 was supported by the data.

### Limitations and Strengths

The BSAS needs to be further evaluated in future studies, as it has only been investigated in the present cross-sectional study. As such, the results may have been influenced by the common method bias, creating inflated relationships between study variables (Podsakoff et al., 2003). There was a preponderance of females in the sample. However, gender was included as an independent variable in the regression analyses and thus was adjusted for in terms of the multivariate relationship between study variables. Self-selection may also have influenced the results since participants responded to an online newspaper article about excessive shopping, perhaps attracting certain groups such as younger people and excessive users of internet shopping. However, the latter may also be viewed as a strength since having people with shopping problems in the sample could strengthen the validity of the scale in clinical contexts. Still, more studies examining the psychometric properties of the BSAS are crucial. It should also be noted that some of the deleted items had just marginally lower loadings than the retained items.

Another limitation that should be acknowledged is that the present study utilized an online survey. It is known that online shoppers may differ in some key ways so the sample may carry some bias in that regard (Wang and Yang, 2008; Weinstein et al., 2015). However, web-based data is applicable given that there is little empirical evidence that such results would be skewed (Pettit, 2002; Gosling et al., 2004). Also, when comparing the data from the present study with data from nationally representative oﬄine samples concerning overlapping instruments, such as the Mini-International Personality Item Pool (Mini-IPIP), similar results are found (Andreassen et al., 2014) suggesting that the present sample is not extremely deviant/skewed. Still, the BSAS also requires validation in other cultures. More specifically, the test-retest reliability should be investigated. Also, some important validated scales were not chosen for comparison to BSAS [e.g., the Compulsive Buying Scale (CBS), Faber and O’Guinn, 1992]. This may be a limitation as well.

However, the very large sample size represents one of the study’s key strengths and is an asset in providing high statistical power to the analyses carried out. Other strengths of the present paper are the use of a strict statistical procedure to finalize the scale and the use of relevant constructs and validated instruments in the validation process. In addition, the BSAS is a generic shopping addiction tool, rather than focusing on specific consumer patterns (e.g., what happens when one enters a shopping center, or what credit one uses such as checks, credit card, or cash). Therefore, the BSAS may be used for measuring both online and oﬄine shoppers and is therefore more in synch with current shopping patterns. Another strength of the study was that the survey was administrated in nationwide newspapers, and not local ones. These national newspapers are also known for having very different reader groups. Hence, the sample probably represents a wide range of Norwegian people and may be more representative than other studies that have used self-selected samples.

The BSAS was constructed simply by taking the highest item from each of seven 4-item clusters, with each item receiving equal weight in the total BSAS score and in accord with clinical practice (American Psychiatric Association, 2013). This provides representation of all domains considered important to the addiction construct and thus theoretically robust. However, it is possible given the unidimensional trait structure of shopping addiction, that alternative item selection might enable better specification of individuals’ scores on the latent trait. As this topic is outside the scope of the present paper, future analyses using modern psychometric approaches could examine individual item sensitivity to this trait and enable an even more efficient computerized adaptive test with equally high precision of measurement.

## Conclusion

The study demonstrated that the BSAS has good psychometrics, structure, content, convergent validity, and discriminative validity, which should encourage researchers to consider using it in epidemiological studies and treatment settings. Although this study did not attempt any cut-off evaluation for classification of shopping addicts, such classification could be conducted using traditional cut-off approaches. For other addictive behaviors, a polythetic procedure is normally used (e.g., whereby endorsing around 50% of the total set of criteria is enough to be classified as an addict). For example, pathological gambling is diagnosed when five out of the 10 criteria are met (American Psychiatric Association, 2013). In line with this, a tentative cut-off score for BSAS may involve the scoring of 3 (≪agree≫) or 4 (≪completely agree≫) on at least four of the seven items. However, this should be investigated further. Given this, the BSAS may be freely used by researchers in their future studies in this field.

## Author Contributions

CA led the conception and design of the study, the literature search, analysis, interpretation of the data, drafting, writing, and revising the work. All authors contributed to the design (MG, SP, TT), analysis (SP), interpretation of data (MG, SP, RB, TT, EA), and/or writing and revising the work critically for important intellectual content (MG, SP, RB, TT, EA). All authors read and approved the final version of the work to be published (CA, MG, SP, RB, TT, EA), and agreed to be accountable for all aspects of the work in ensuring that questions to the accuracy of any part of the work are appropriately investigated and resolved (CA, MG, SP, RB, TT, EA).

## Conflict of Interest Statement

The authors declare that the research was conducted in the absence of any commercial or financial relationships that could be construed as a potential conflict of interest.
